# Tumor of the Choroid

**Published:** 1867-09

**Authors:** 


### TUMOR OF THE CHOROID.

Dr. Holmes exhibited an eye of a patient at the Chicago Charitable Eye and Ear Infirmary, 52 years of age, who had suffered two years most violent pain in and around the orbit. The globe was soft to the touch, the pupil occluded, iris discolored, conjunctiva congested, but not œdematous; vision had long been totally extinct. The diagnosis was doubtful. As all efforts had failed to relieve the pain, it was deemed best to extirpate the globe. Within it was found a well defined, hard, very dark colored tumor, three-eighths of an inch in diameter, almost spherical in form, attached to the choroid, at its outer and posterior portion, near the optic nerve. It was composed of a fine granular mass, with pigment cells.

